# Dental implant placement with inferior alveolar nerve repositioning in severely resorbed mandibles: a retrospective multicenter study of implant success and survival rates, and lower lip sensory disturbances

**DOI:** 10.1186/s40729-021-00334-x

**Published:** 2021-06-09

**Authors:** George Deryabin, Simonas Grybauskas

**Affiliations:** 1 Chicago, USA; 2 S’OS Orthognathic Surgery, Vytenio 22–201, Vilnius, Lithuania

**Keywords:** Inferior alveolar nerve transposition, Inferior alveolar nerve lateralization, Dental implant survival rate, Dental implant success rate, Neurosensory disturbance

## Abstract

**Background:**

The purpose of this study was to analyze medium-to-long-term implant success and survival rates, and lower lip sensory disturbance after placement of dental implants with simultaneous inferior alveolar nerve (IAN) repositioning.

**Methods:**

Fifteen patients (3 men, 12 women) treated in two centers were included in this retrospective study. The ages of the participants ranged from 19 to 68. A total of 48 dental implants were placed in 23 posterior mandibular segments simultaneously with IAN transposition or lateralization. The residual bone above the IAN ranged from 0.5 to 7.0 mm. Crestal bone changes were measured using cone beam computed tomography (CBCT) images. Disturbance of the IAN was evaluated subjectively using a modified questionnaire.

**Results:**

The healing process was uneventful in fourteen patients. In one patient, spontaneous fracture of the operated mandible occurred on tenth day after the surgery. The implant in the fracture line was removed at the time of open reduction and fixation. One more implant was lost after 5 years of loading. Therefore, the overall dental implant survival rate was 95.8%, whereas all implants in function were judged as successful after a follow-up period of 1 to 10 years. Transient neurosensory disturbances (ND) were observed in all patients who underwent IAN lateralization and IAN transposition. At follow-up times of 3 years, 5 years, and 10 years, weak hypoesthesia remained in two subjects treated with IAN transposition. None of the patients developed neuropathic pain after the procedure.

**Conclusions:**

Within the limitations of this study, we conclude that reconstruction of severely resorbed mandibles with dental implants in conjunction with IAN repositioning is an effective and reliable technique. Although neurosensory disturbances are the most common complication after surgery, they tend to resolve over time. Advanced surgical skills are required to perform this technique.

## Background

Implant placement in severely resorbed posterior mandibles (classes V and VI of Cawood-Howell classification) is challenging because of the insufficient bone volume, diminished amount of attached and unattached mucosa, and superficial location of the inferior alveolar nerve [1]. Several treatment strategies have been proposed in the literature to address this problem including placement of short implants, vertical ridge augmentation, sandwich osteotomy, and distraction osteogenesis. Another option is to expose the IAN, displacing it laterally from the canal with nerve lateralization or transposition [2, 3].

The main advantages of this technique are:

1. Dental implants are inserted simultaneously in a single procedure. Therefore, it reduces the number of surgeries, overall cost of treatment, and shortens treatment time [4].
2. Dental implants are placed in native bone, eliminating the need for vertical ridge augmentation, which may be subject to resorption over years [5, 6].
3. This is an appropriate technique for situations where the IAN is located in the superior part of the body of the mandible, and even mild atrophy of the edentulous alveolar process will bring the alveolar ridge within close proximity of the neuro-vascular bundle. The incidence of “high” IAN locations ranges from 14.6 to 30.7% [7, 8].

**Fig. 1:**
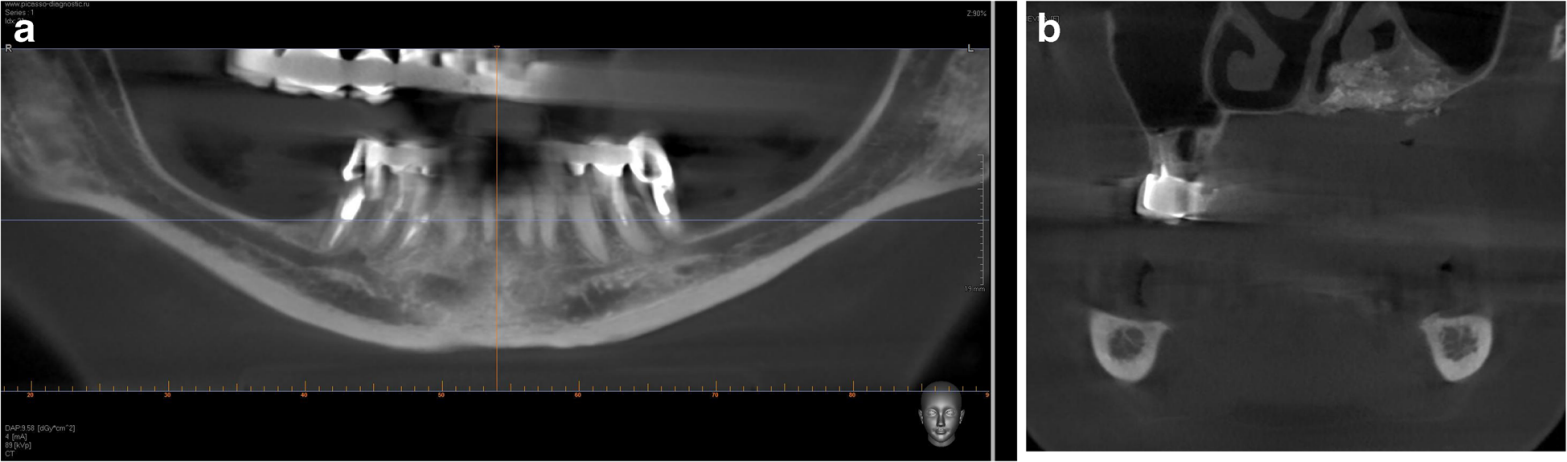
CBCT scan before surgery. **a** A panoramic view. **b** A coronal view

**Fig. 2:**
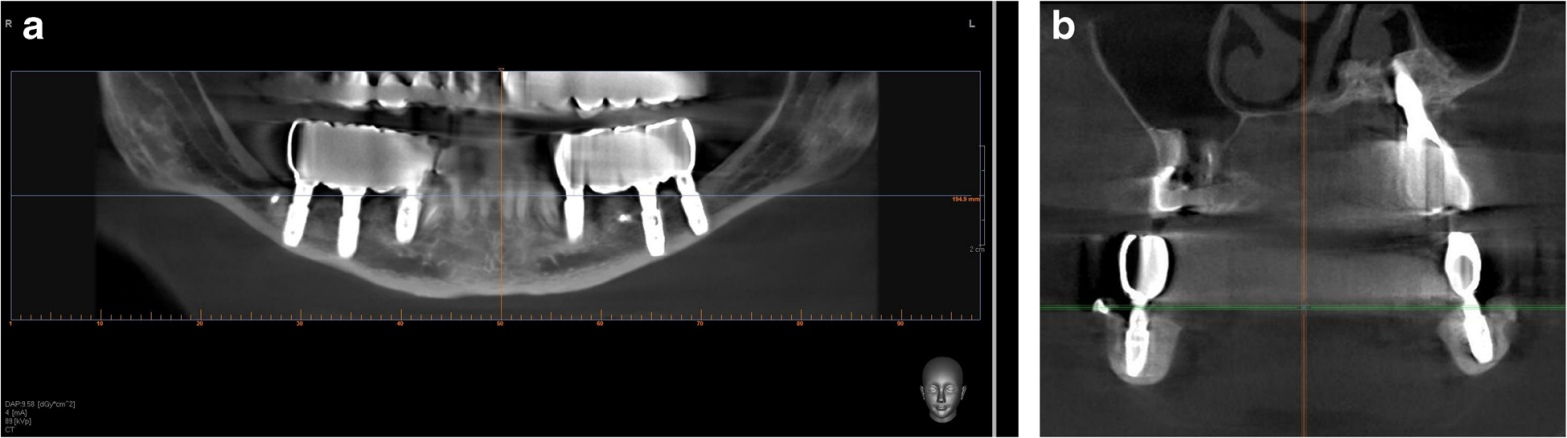
CBCT scan 2 years after surgery. **a** A panoramic view. **b** A coronal view

**Fig. 3:**
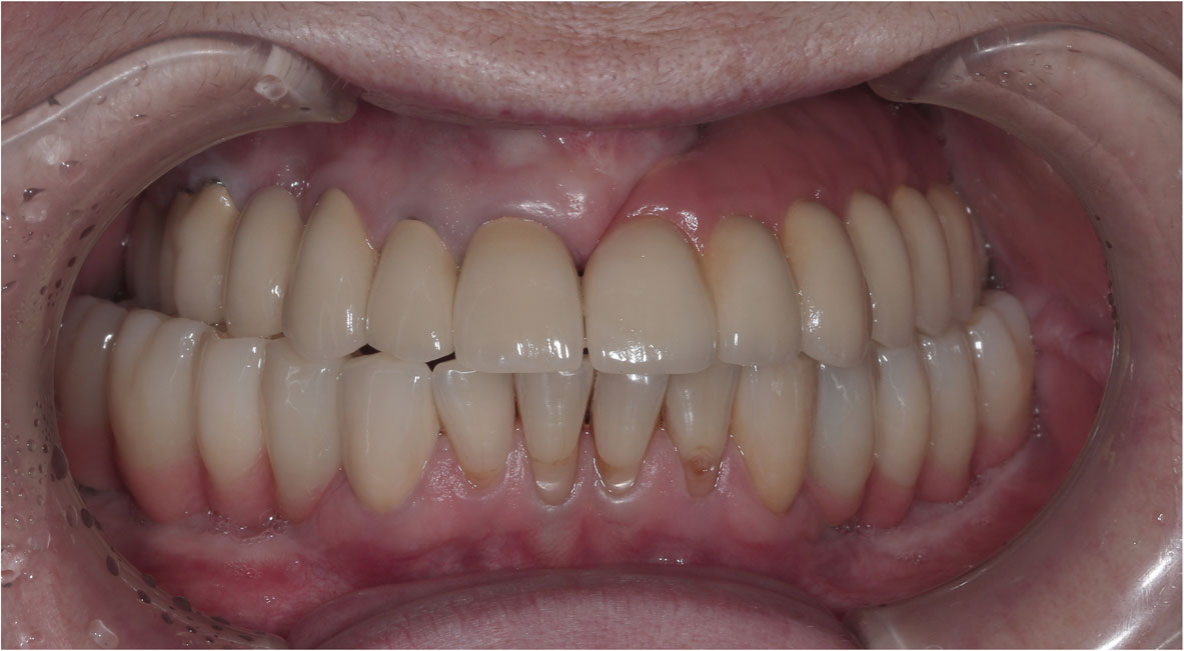
The intraoral view of the final restoration. Notice bilateral posterior crossbite. (Prosthodontist: Renat Aubov)

However, there are some limiting factors and disadvantages of the technique:

1. IAN lateralization/transposition requires advanced skills in surgical nerve handling and management of complications [9].
2. The risk of specific complications, such as fracture of the mandible and damage of the IAN at the time of repositioning, or during vestibuloplasty in the second stage [9–11].
3. Due to the transverse pattern of bone atrophy, the maxillary arch becomes narrower and the mandibular arch becomes broader [1]. In such a jaw relationship, dental implants have to be placed and restored in posterior crossbite in order to decrease axial loading and reduce the shear stress on retaining screws [12, 13]. (Figs. 1, 2, 3)

The purpose of this study was to analyze medium-to-long-term implant success and survival rates, and lip sensory disturbance after placement of dental implants in combination with IAN repositioning.

## Methods

**Fig. 4:**
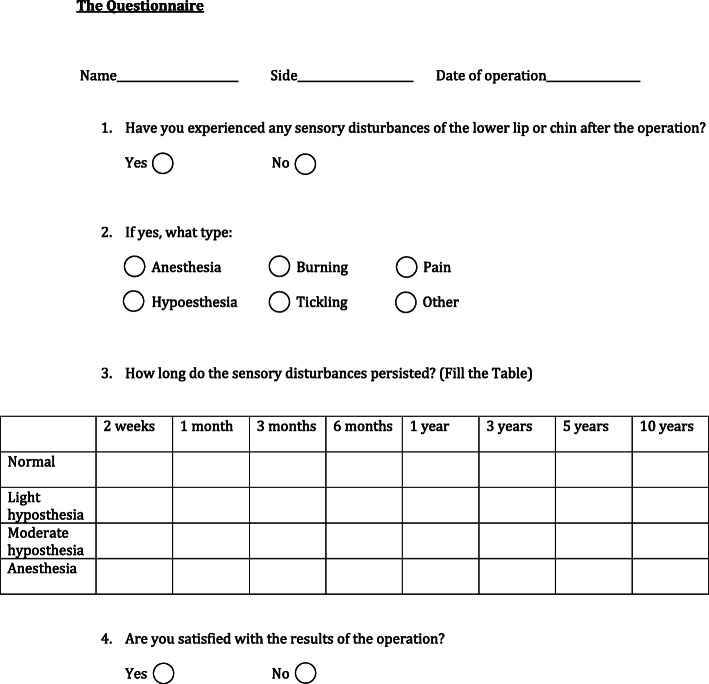
A questionnaire to register neurosensory disturbances after the operation

The authors conducted a retrospective evaluation of the treatment results of 15 patients with bone defects of the posterior mandible who underwent implant placement with IAN repositioning in two centers from December 2009 to September 2019. Bone height above the mandibular canal was assessed using CBCT, and all mandibles were ranked as class V or class VI according to Howell-Cawood classification. Subjects included in the study were free of active infections, insulin-dependent diabetes, and had no history of radiotherapy, chemotherapy, or oral cancer surgery. All patients provided written informed consent and received a modified questionnaire proposed by Hashemi HM [14] to register any neurosensory disturbances (ND) after the operation (Fig. 4).

**Fig. 5:**
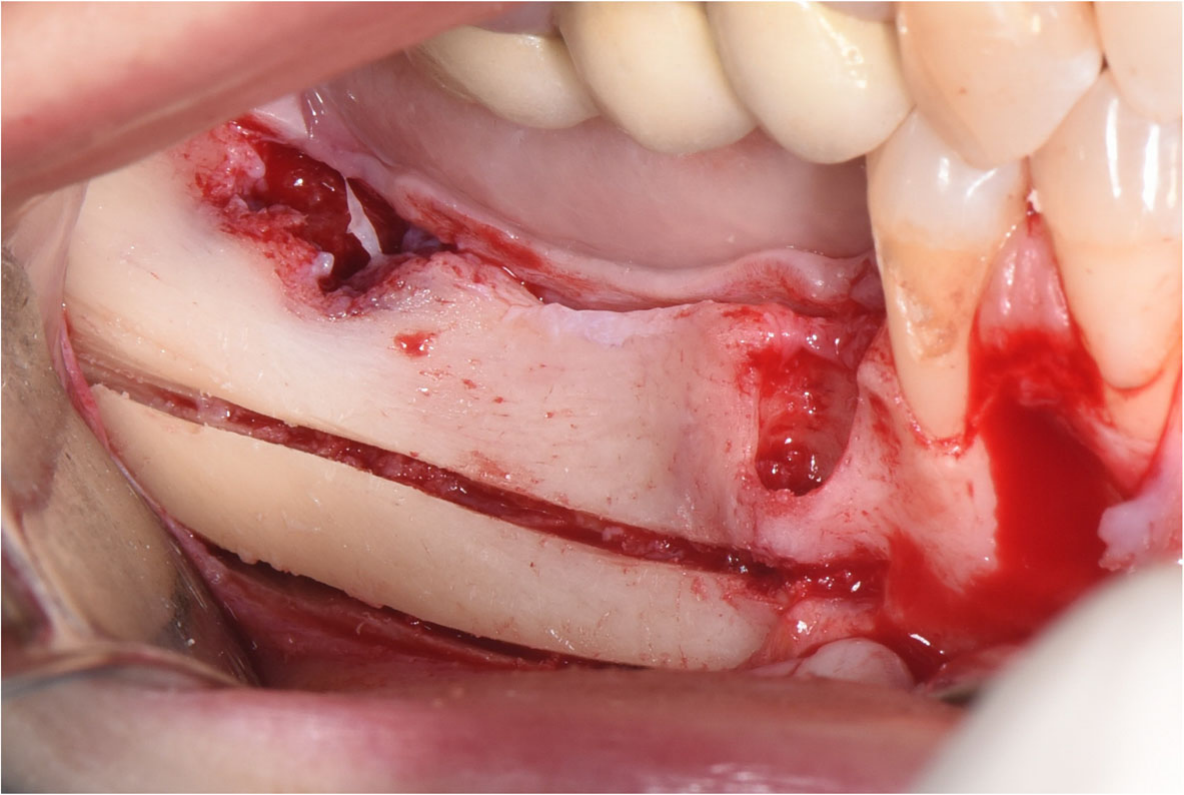
A full corticotomy performed with a piezoelectric saw creating a rectangular bony window lateral to the inferior alveolar canal

**Fig. 6:**
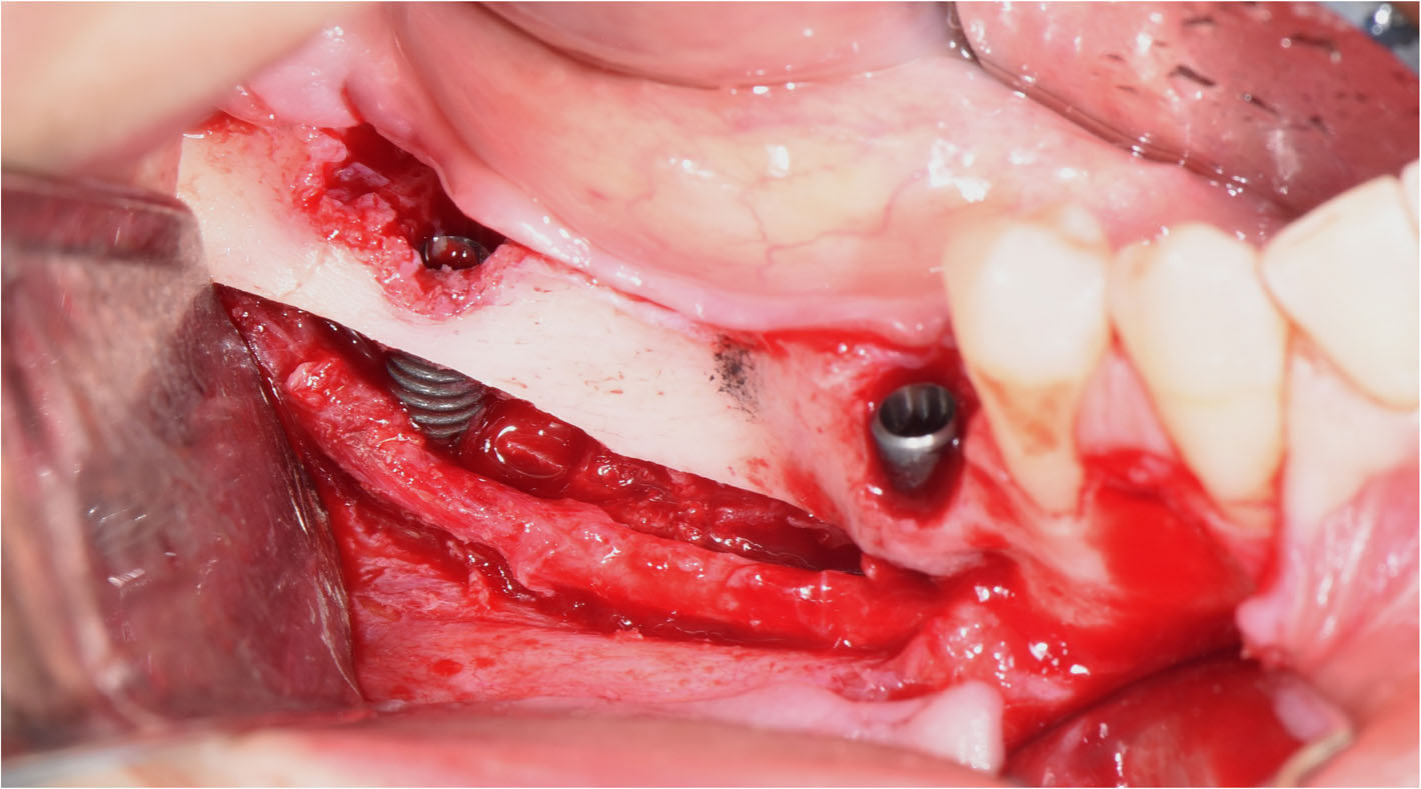
The neurovascular bundle is mobilized from the canal and retracted laterally while the implants are installed

**Fig. 7:**
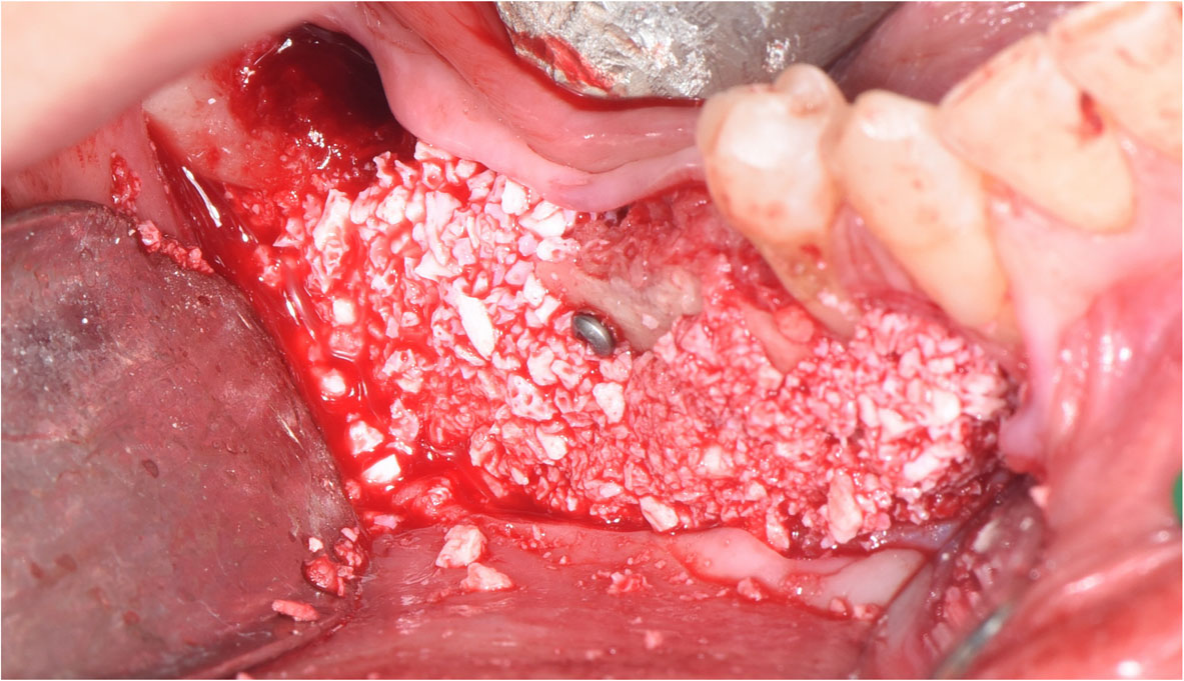
Additional horizontal bone augmentation is performed by means of GBR

**Fig. 8:**
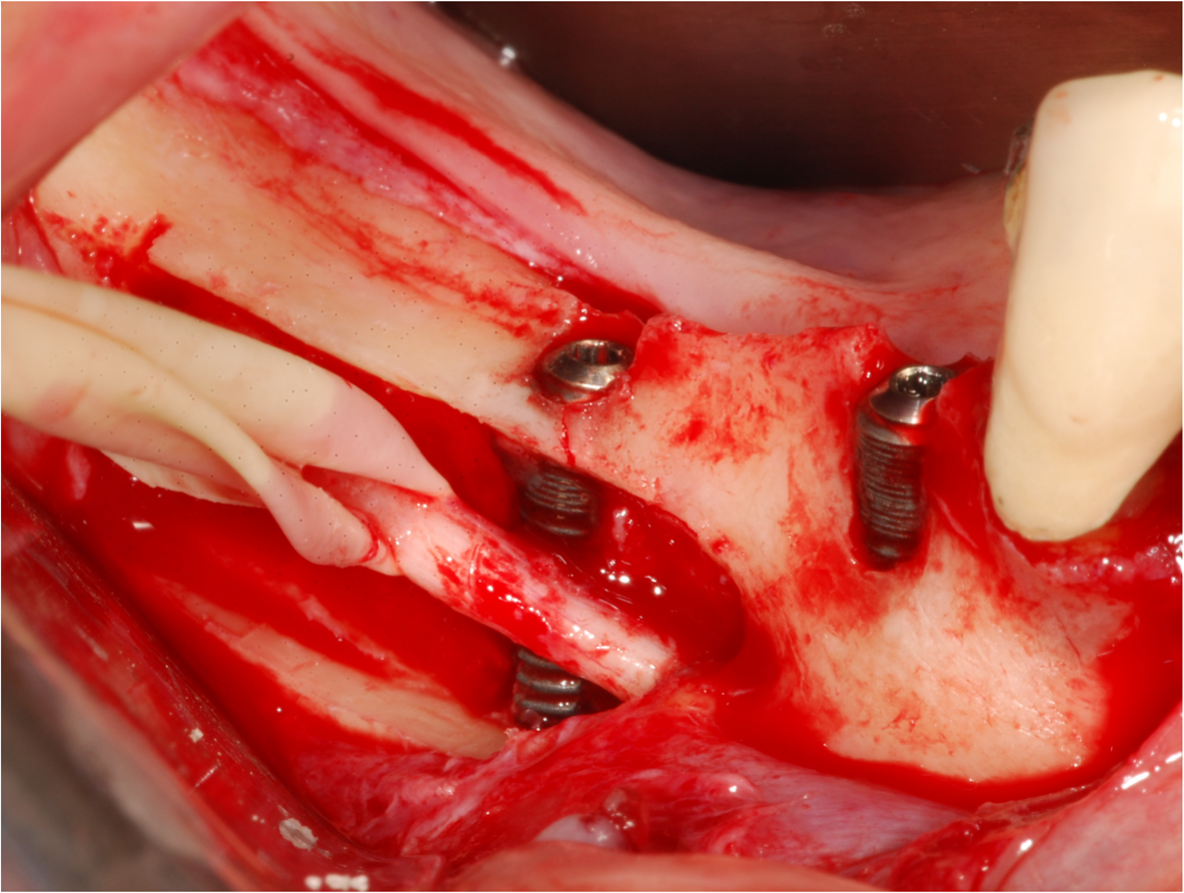
Another case of nerve lateralization. The neurovascular bundle is mobilized from the canal and retracted laterally while the implant is installed

**Fig. 9:**
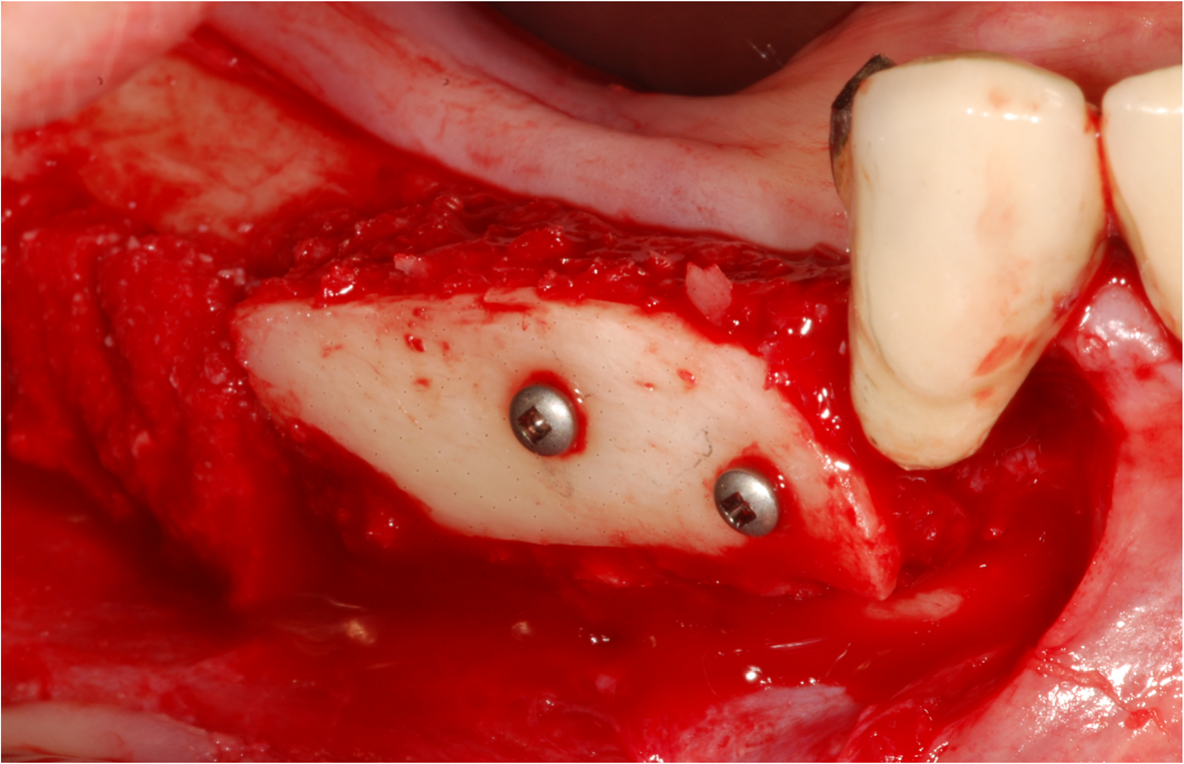
Additional horizontal bone augmentation is performed by means of autogenous onlay block graft. The cortical bony window (after creating access to the IAN) is utilized as a source of autogenous bone

**Fig. 10:**
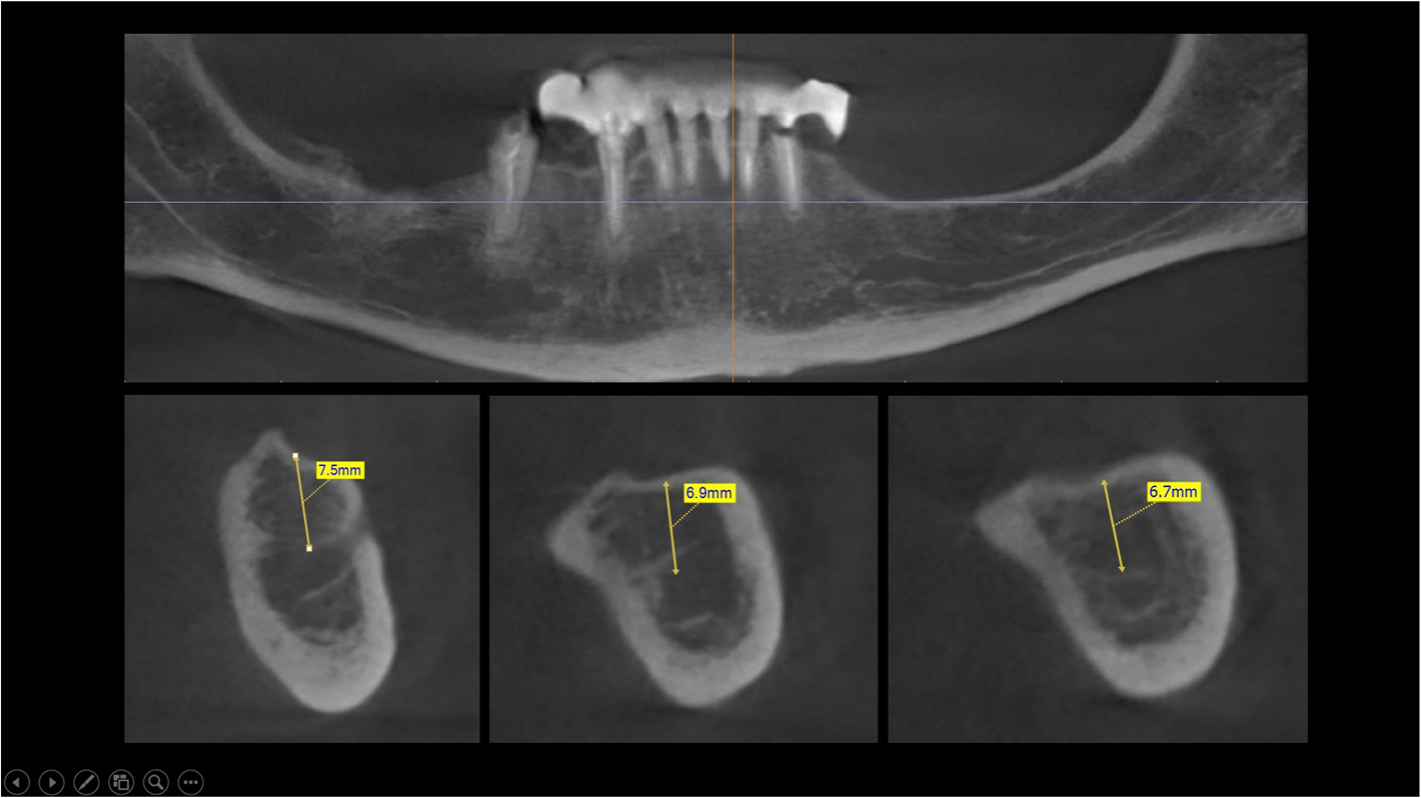
CBCT scan before surgery. A panoramic and cross-sectional view

**Fig. 11:**
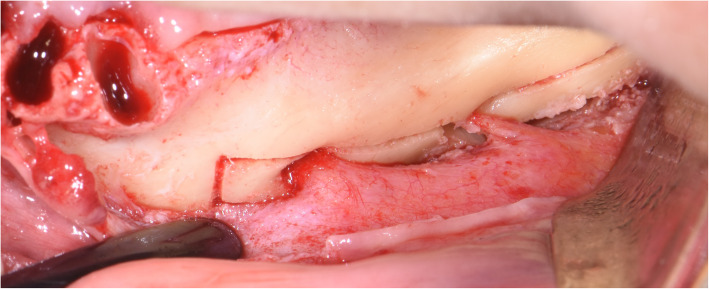
A full corticotomy performed with a piezoelectric saw creating a rectangular bony window lateral to the inferior alveolar canal. The osteotomy window was extended anteriorly to expose the mental foramen and incisive branch

**Fig. 12:**
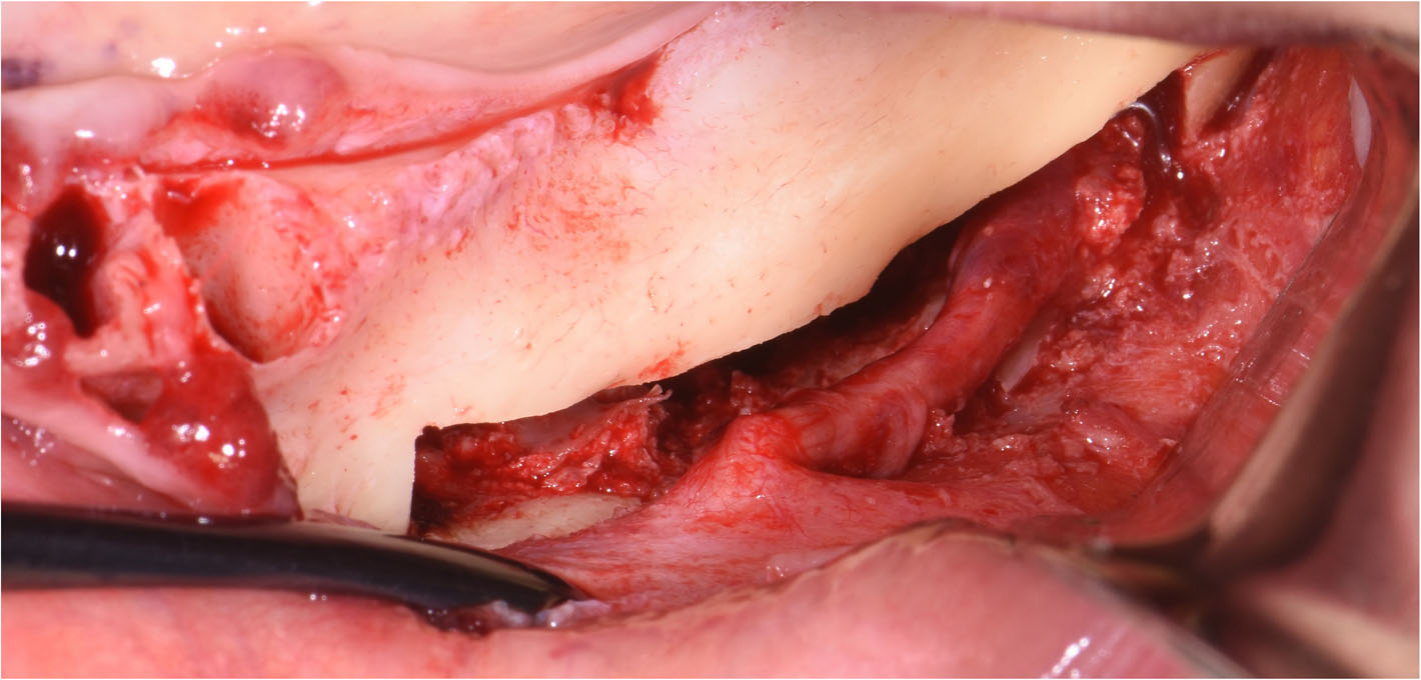
The bony window is removed and the neurovascular bundle is mobilized from the canal. The incisive branch is severed, and the IAN freed from the canal and moved laterally

**Fig. 13:**
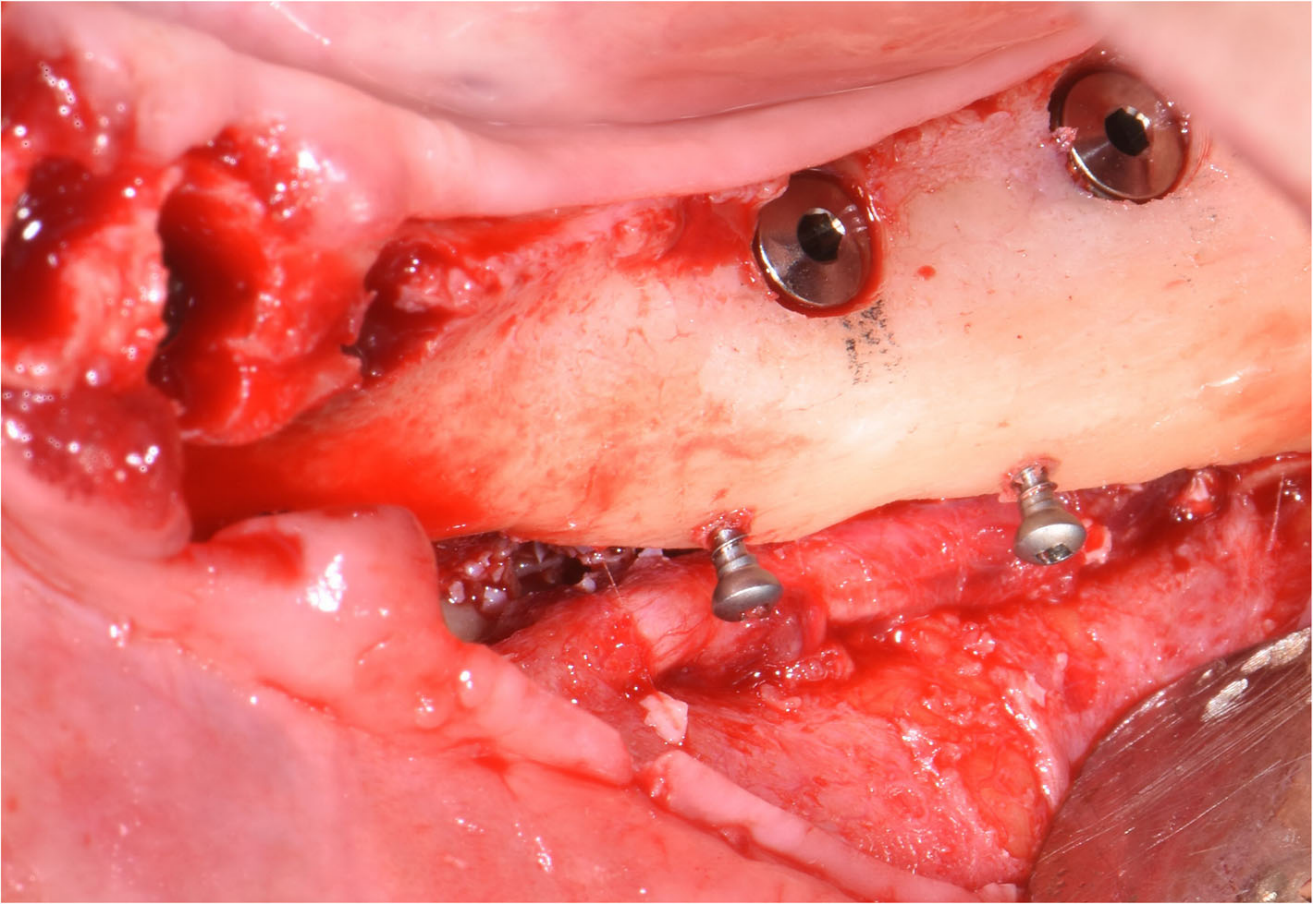
The neurovascular bundle is mobilized from the canal and retracted laterally while the implants are installed. Two titanium miniscrews are placed halfway on the lateral side of the mandible at the osteotomy lines to maintain the displaced neurovascular bundle in a new position and indicate its new location

**Fig. 14:**
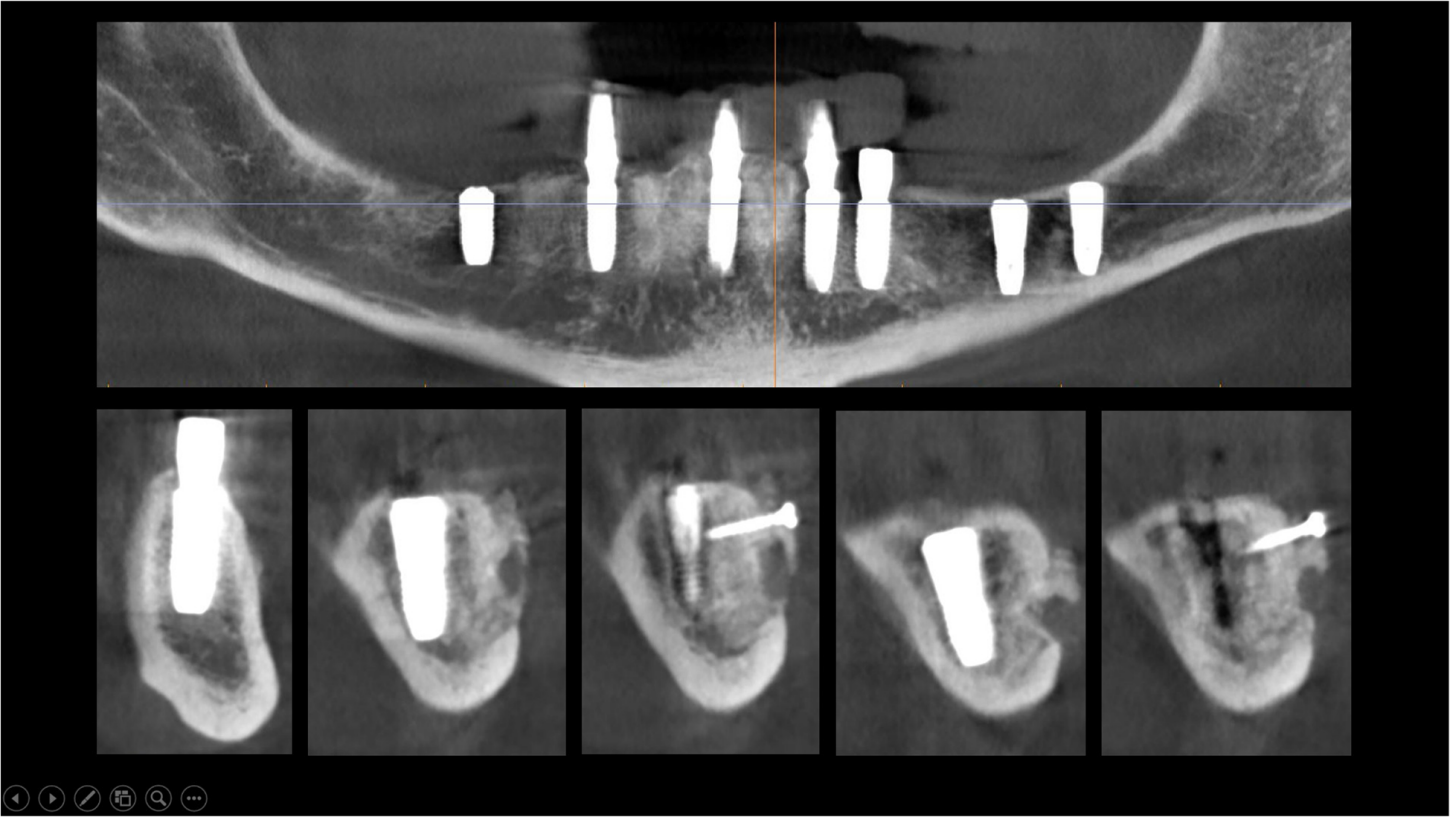
CBCT scan after surgery. A panoramic and cross-sectional view

**Fig. 15:**
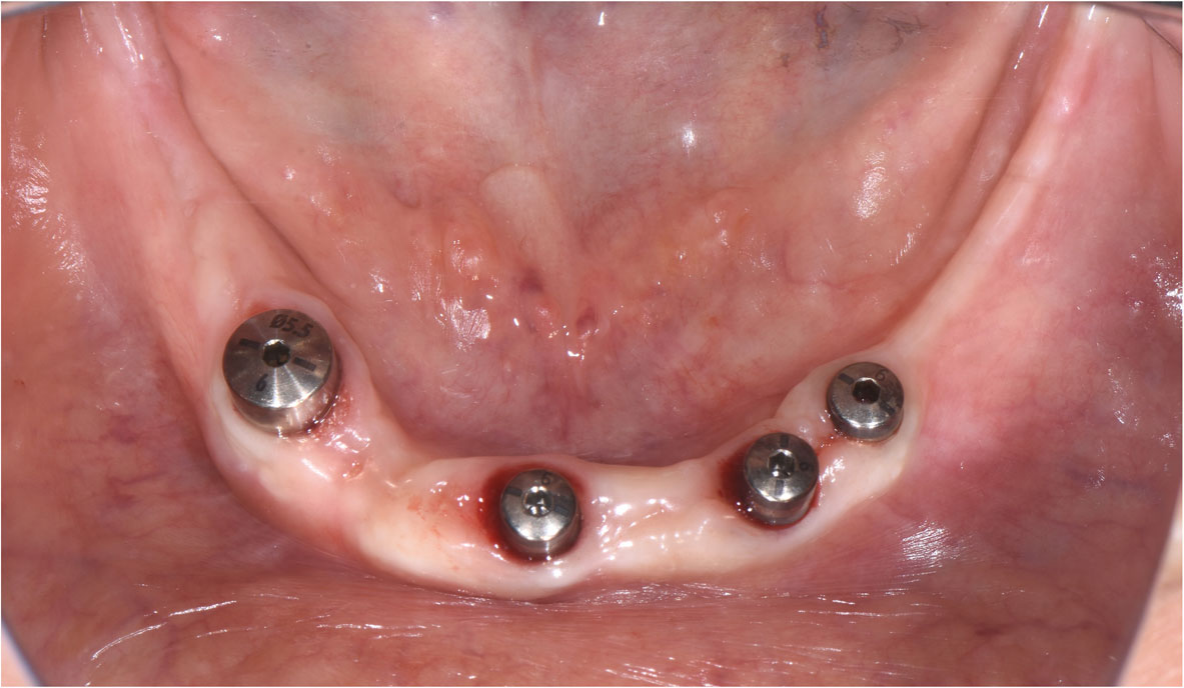
Intraoral view at the time of second-stage surgery. Notice a lack of keratinized gingiva

**Fig. 16:**
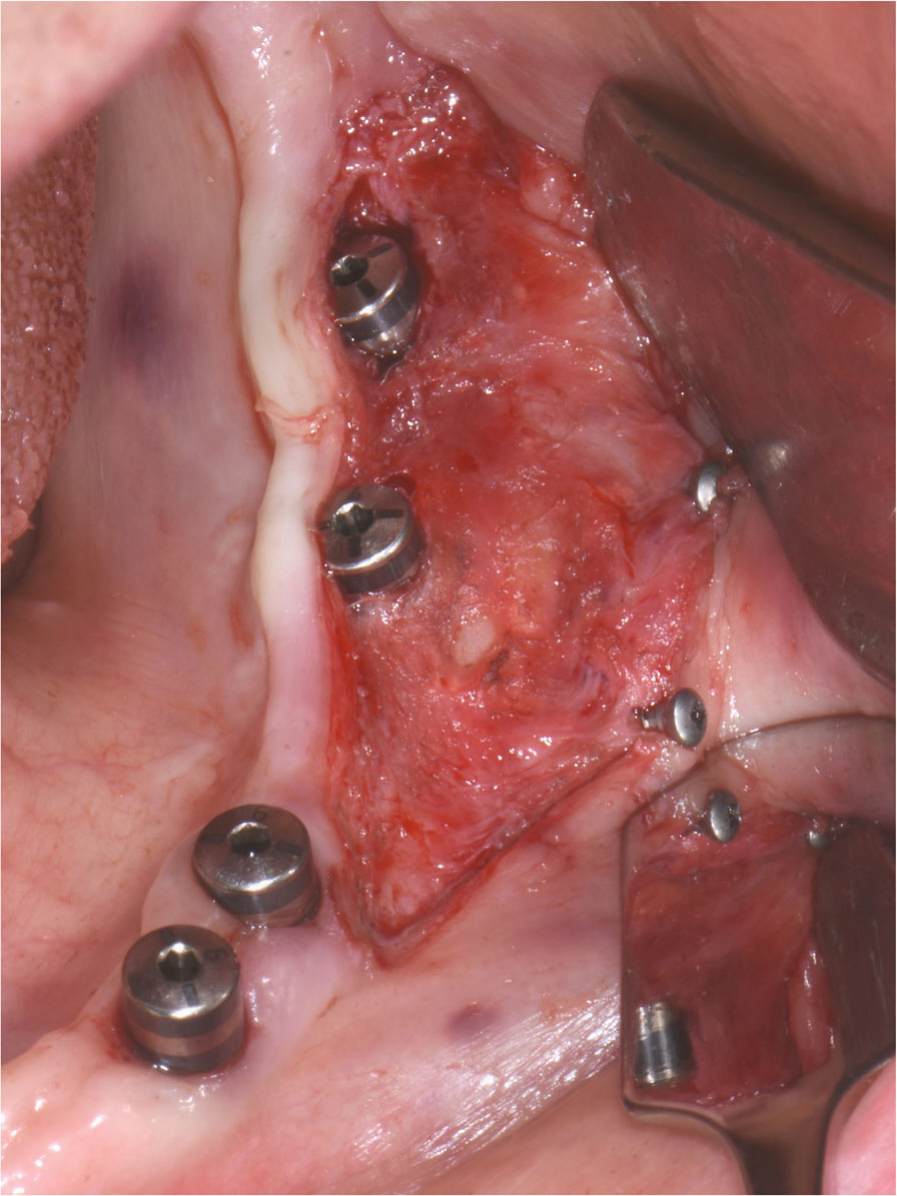
A split thickness flap is separated exposing the periosteum. Two titanium miniscrews indicate the new location of the displaced IAN

**Fig. 17:**
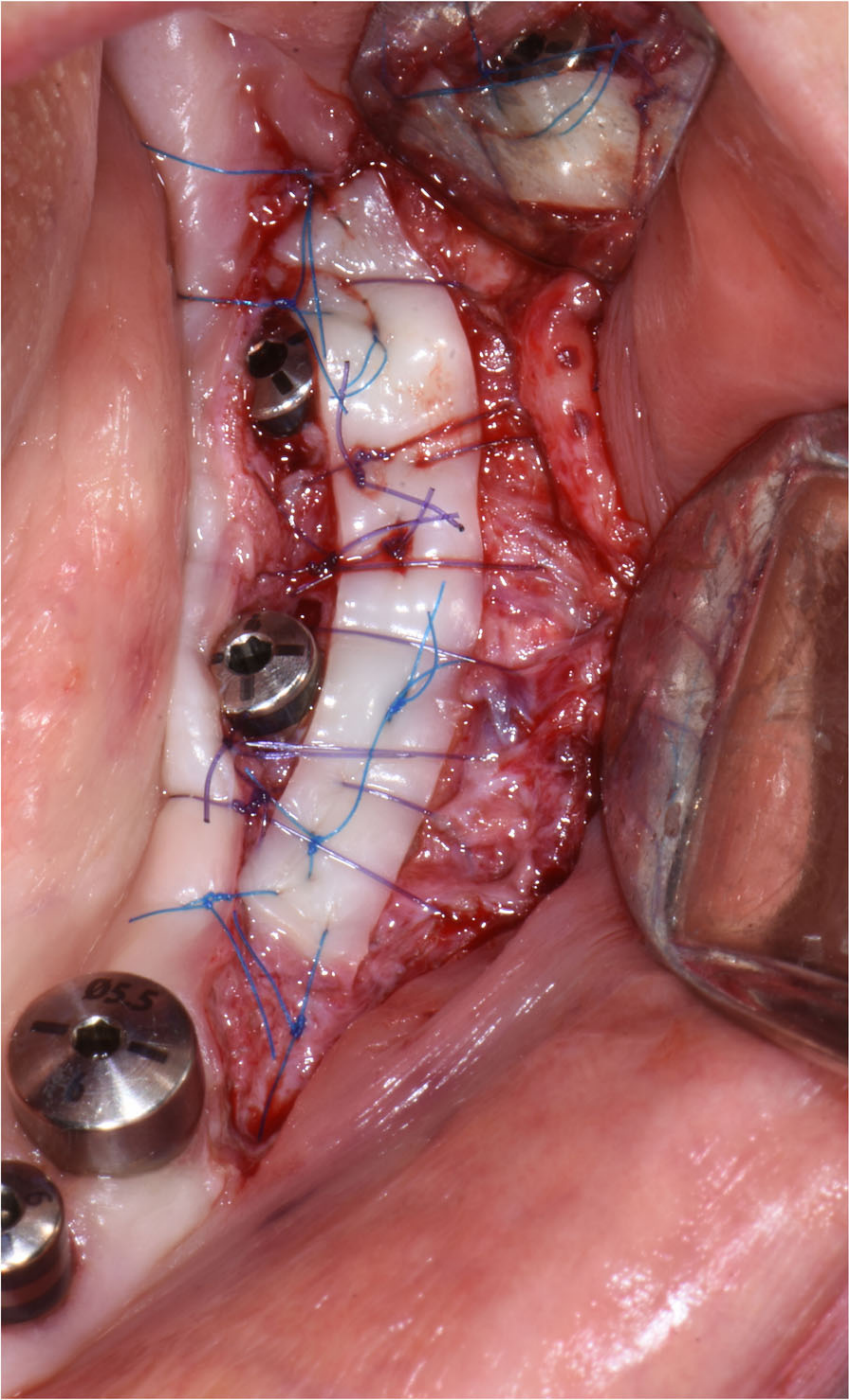
Vestibuloplasty with free gingival graft is performed

**Fig. 18:**
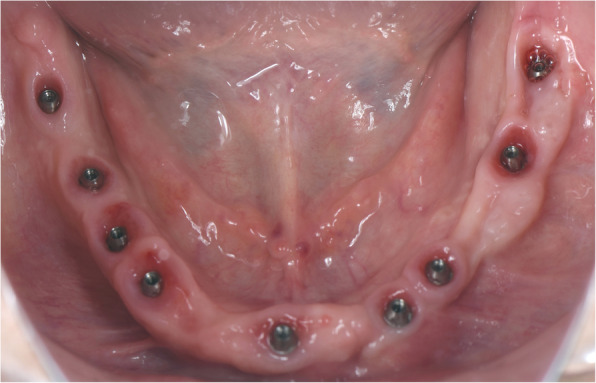
Intraoral view at the time of uni abatment placement

**Fig. 19:**
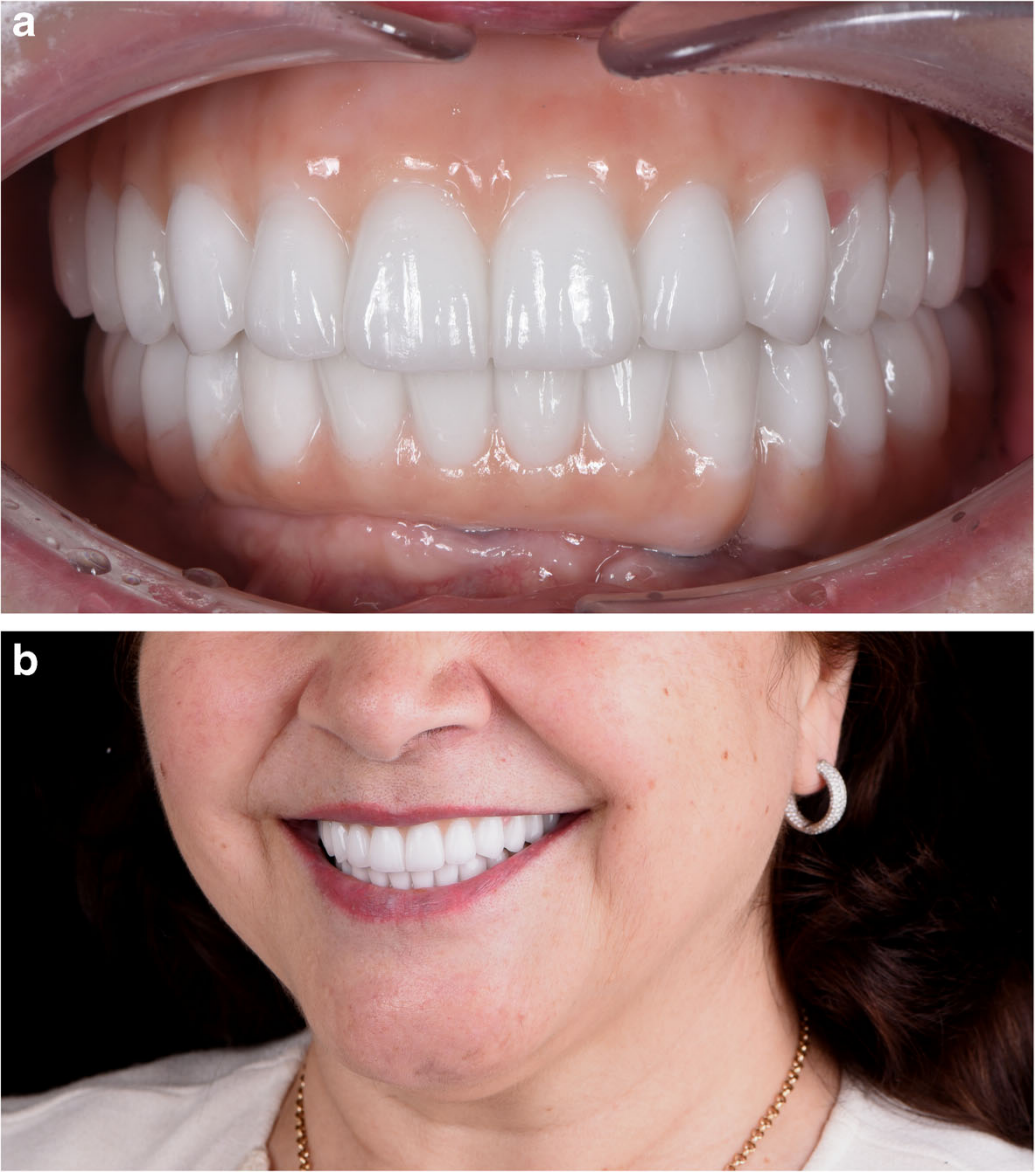
**a**, **b** Final restoration. (Prosthodontist: Evgeniy Shor)

**Fig. 20:**
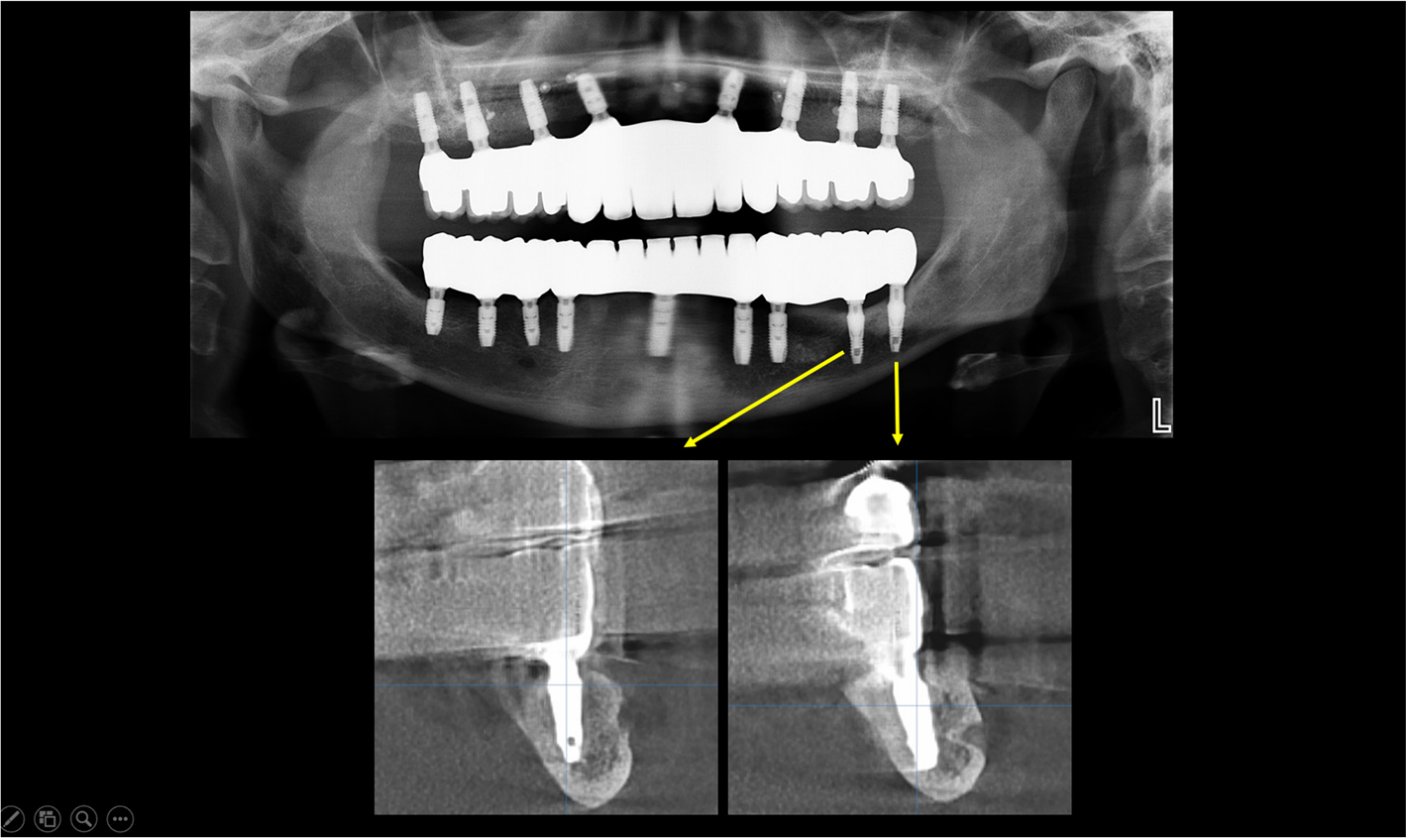
CBCT scans 2 years after the delivery of the final prothesis

The surgical protocol and postoperative medications were standardized across the participating centers. The operations were performed under a combination of local anesthesia and intravenous sedation. A triangular mucoperiosteal flap, as well as an envelope flap (without a vertical releasing incision), were used to expose the alveolar crest and lateral body of the mandible of the edentulous area. A full corticotomy was performed with a piezoelectric saw creating a rectangular bony window lateral to the inferior alveolar canal. The bony window was removed by exposing the inferior alveolar canal. The neurovascular bundle was mobilized from the canal and retracted laterally while the implants were installed (Figs. 5, 6, 7, 8, and 9). In cases of nerve transposition (distalization), the osteotomy window was extended anteriorly to expose the mental foramen and incisive branch. The incisive branch was then severed, and the IAN was freed from the canal and moved laterally. In certain patients with “high” IAN locations combined with considerable bone resorption, titanium miniscrews were placed halfway on the lateral side of the mandible at the osteotomy lines to maintain the displaced neurovascular bundle in a new position and indicate its new location [11] (Figs. 10, 11, 12, 13, 14, 15, 16, 17, 18, 19, and 20). The space between the implants and neuro-vascular bundle was grafted, and the muco-periosteal flaps were repositioned and sutured with resorbable sutures. Additional horizontal bone augmentation was performed in five cases simultaneously with IAN repositioning and implant placement by means of guided bone regeneration (GBR) or autogenous onlay block grafts (Figs. 7 and 9). In all cases, cortical bony windows (after creating access to the IAN) were utilized as a source of the autogenous bone for bone augmentation. The healing time before the second stage was 6 months. The function of the IAN was evaluated according to patients’ subjective reports 2 weeks, 1 month, 3 months, 6 months, 1 year, 3 years, 5 years, and 10 years after the operation. Changes in the peri-implant crestal bone level were measured using CBCT images during follow-up visits for a period of 1 to 10 years.

## Results

Overall, 23 IAN repositioning surgical procedures with simultaneous implant placement were performed in 15 patients (12 women, 3 men). Among them, 17 patients underwent IAN lateralization and 6 patients underwent IAN transposition. Patient ages ranged from 19 to 68 years. A total of 48 dental implants were installed. The mean residual bone height above the IAN was 4.3 mm (range, 0.5 to 7.0 mm). The mean follow-up time was 5.1 years (range, 1–10 years). One implant was removed due to spontaneous mandibular fracture 10 days after surgery, and one more implant was lost after 5 years of loading. Therefore, the implant survival rate was 95.8%, and the implant success rate, according to Clementini’s [15] criteria, was also 95.8%. All patients reported transient numbness during the first 2 weeks after surgery. By the end of the third month, only three patients experienced moderate mental nerve hypofunction. After 1 year, two subjects reported weak hypoesthesia. At the 3-, 5-, and 10-year follow-ups, weak hypoesthesia remained in 2 subjects treated with IAN transposition (Tables 1 and 2). According to the survey, all patients were completely satisfied with the results of the operation.

**Table 1:** Evaluation of patient sensitivity alterations after IAN transposition

	2 weeks	1 month	3 months	6 months	1 year	3 years	5 years	10 years
Normal	0	0	2	3	4	4	2	n/a
Light hyposthesia	0	4	2	1	2	2	0	n/a
Moderate hyposthesia	5	2	2	2	0	0	0	n/a
Anesthesia	1	0	0	0	0	0	0	n/a
Total sites	6	6	6	6	6	6	2	n/a
Total patients	4	4	4	4	4	4	1	n/a

**Table 2:** Evaluation of patient sensitivity alterations after IAN lateralization

	2 weeks	1 month	3 months	6 months	1 year	3 years	5 years	10 years
Normal	1	2	14	14	14	17	8	1
Light hyposthesia	4	13	3	3	3	0	0	0
Moderate hyposthesia	10	2	0	0	0	0	0	0
Anesthesia	2	0	0	0	0	0	0	0
Total sites	17	17	17	17	17	17	8	1
Total patients	11	11	11	11	11	11	6	1

## Discussion

Rehabilitation of severely resorbed posterior mandibles with dental implants can be performed using two different strategies: vertical bone augmentation in order to gain alveolar height for longer implants, or utilizing the residual native bone by placing either short implants or longer implants simultaneously with IAN repositioning.

The success of bone augmentation largely depends on the blood supply to the recipient site, quality of the soft tissues, availability of autogenous bone, and/or growth factors, age, and health status of the patient. The amount of osteoprogenitor cells in the bone marrow, and as a consequence, the osteogenic potential of the autogenous bone declines dramatically with age [16–18]. Moreover, many patients with severe bone defects have a previous negative dental implant experience. Therefore, the blood supply and quality of soft tissues in these areas are impaired, and the availability of the bone for harvesting is limited. All the abovementioned factors can negatively affect bone regeneration. In addition, the total cost of bone augmentation could be significantly higher than IAN repositioning, especially when growth factors or additional autogenous bone-graft donor sites are utilized. As a result, IAN repositioning with dental implant placement is a valuable option for patients whose medical condition or physiology (age, etc.) necessitates a reduction in the number or extensiveness of surgical steps in order to achieve stable, functional results following treatment.

Short implants are an acceptable alternative in patients with lowered bone height in the posterior mandibles. However, it is often challenging to place them without damaging the IAN when the bone height is less than 8 mm above the canal. In our study, the mean residual bone height above the IAN was 4.3 mm (range, 0.5 to 7.0 mm). Another limiting factor is poor bone quality in the posterior segments of severely resorbed mandibles due to osteoporosis. Therefore, it may be extremely difficult to achieve monocortical primary stability of short dental implants at the time of installation, which is one of the most important criteria for successful osseointegration [19].

In contrast, poor bone quality in combination with extensive resorption poses a serious risk of rare potential complications such as mandibular fracture, as IAN repositioning with implant placement does not restore the alveolar ridge anatomy, but rather weakens the basal bone [4, 20, 21].

**Fig. 21:**
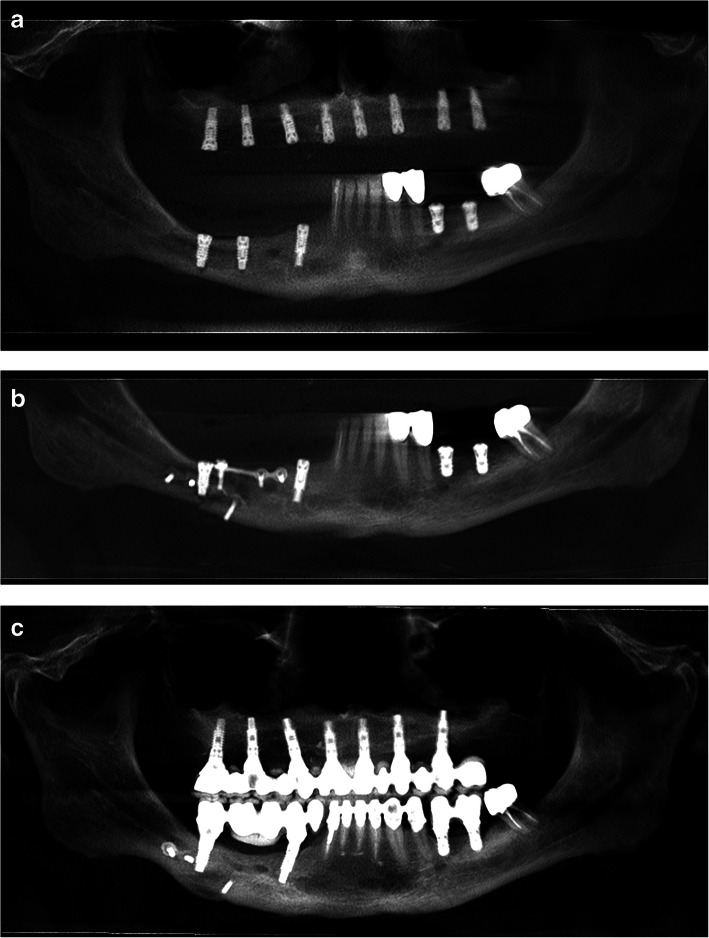
**a** A panoramic view of CBCT scan after IAN lateralization with implant placement. **b** A panoramic view of CBCT scan after open reduction and fixation of the mandible. **c** A panoramic view 1 year after the delivery of the final prothesis

One patient in our study experienced a spontaneous fracture of the mandible while yawning 10 days after surgery. The implant in the fracture line was removed at the time of open reduction and fixation. After 2 months, two implants on both sides of the fracture line were used for final prosthetic rehabilitation, which also acted as reinforcement to fix the mandibular fracture (Fig. 21a–c).

Some authors believe that surgery of the neurovascular bundle changes the blood supply to the lateral mandible and consider it as the main reason for delayed bone fractures. However, as the inferior alveolar artery mainly supplies the teeth of the mandible, gingivae, and the skin over the chin and lower lip, its role in the blood supply to the lateral mandible is not critical [22, 23]. Moreover, with the increase of age and with the loss of teeth, the importance of the inferior alveolar artery in blood perfusion of the lower jaw decreases, and the blood supply to the mandible mostly depends on the periosteum [22, 24, 25]. We suppose that the fracture on tenth day after surgery occurred due to loss of bone structural integrity after surgical trauma. Our assumptions coincide with other reports in the literature on the same subject [26–30].

Regardless of the potential risks and in light of the aforementioned discussion, IAN transposition or lateralization in combination with dental implant placement is an attractive treatment modality in cases of severely resorbed posterior mandible. Although postoperative neurosensory disturbances are the most common adverse effects of this procedure, they resolved over time, and none of the patients developed neuropathic pain after the procedure. The diagnosis of neurosensory dysfunction of the IAN is based on subjective clinical sensory testing and objective sensory tests. According to Loescher et al. [31], a patient’s subjective report is the most sensitive measure of sensory abnormalities, as objective testing may not diagnose minor ND. Moreover, patient complaints are the main incentive for seeking further recommendations or treatment of their postoperative altered IAN sensation. Therefore, in the present study, the postoperative function of IAN was evaluated subjectively using a modified questionnaire. In our patients, ND after IAN transposition was higher and remained longer than after IAN lateralization, as IAN transposition appeared to be a more invasive procedure. This assumption is supported by systematic reviews of the same subject [4, 10].

## Conclusions

Within the limitations of the present study, we conclude that reconstruction of severely resorbed mandibles with dental implants in conjunction with IAN repositioning is an effective and reliable technique, despite the complexity and risks of specific complications, such as a spontaneous fracture of the mandible. Although the probability of ND is high, in most cases, it resolves within several months after the operation. Patients tolerated the surgery well and indicated that they would go through it again, considering the benefits they received. Advanced surgical skills are required to perform this technique.

## Data Availability

Not applicable.

## Abbreviations

IAN: Inferior alveolar nerve
ND: Neurosensory disturbances
CBCT: Cone-beam computed tomography
GBR: Guided bone regeneration

## References

[1] Cawood JI, Howell RA (1991). Reconstructive preprosthetic surgery. Int J Oral Maxillofac Surg.

[2] Rosenquist BO. Fixture placement posterior to the mental foramen with transpositioning of the inferior alveolar nerve. Int J Oral Maxillofac Implants. 1992;7(1):45–50. 1398823

[3] Jensen O, Nock D (1987). Inferior alveolar nerve repositioning in conjunction with placement of osseointegrated implants: a case report. Oral Surg Oral Med Oral Pathol.

[4] Vetromilla BM, Moura LB, Sonego CL, Torriani MA, Chagas OL (2014). Complications associated with inferior alveolar nerve repositioning for dental implant placement: a systematic review. Int J Oral Maxillofac Surg.

[5] Rocchietta I, Fontana F, Simion M (2008). Clinical outcomes of vertical bone augmentation to enable dental implant placement: a systematic review. J Clin Periodontol.

[6] Esposito M, Grusovin MG, Felice P, Karatzopoulos G, Worthington HV, Coulthard P. The efficacy of horizontal and vertical bone augmentation procedures for dental implants: a cochrane systematic review. In: Chiappelli F, editor. Evidence-Based Practice: Toward Optimizing Clinical Outcomes. Berlin: Springer; 2010:195–218. [cited 2021 Mar 1]. Available from: 10.1007/978-3-642-05025-1_13

[7] Kieser JA, Paulin M, Law B (2004). Intrabony course of the inferior alveolar nerve in the edentulous mandible. Clin Anat.

[8] Heasman PA (1988). Variation in the position of the inferior dental canal and its significance to restorative dentistry. J Dentistry.

[9] Babbush CA (1998). Transpositioning and repositioning the inferior alveolar and mental nerves in conjunction with endosteal implant reconstruction. Periodontology.

[10] Palacio García-Ochoa A, Pérez-González F, Negrillo Moreno A, Sánchez-Labrador L, Cortés-Bretón Brinkmann J, Martínez-González JM, López-Quiles Martínez J (2020). Complications associated with inferior alveolar nerve reposition technique for simultaneous implant-based rehabilitation of atrophic mandibles. A systematic literature review. J Stomatol Oral Maxillofac Surg.

[11] Deryabin G, Grybauskas S (2020). Inferior alveolar nerve repositioning and securing in conjunction with dental implant placement: a technical note. Int J Implant Dent.

[12] Weinberg LA. The biomechanics of force distribution in implant-supported prostheses. Int J Oral Maxillofac Implants. 1993;8(1):19–31. 8468083

[13] Misch CE (1999). Contemporary Implant Dentistry. Implant Dentistry.

[14] Hashemi HM (2010). Neurosensory function following mandibular nerve lateralization for placement of implants. Int J Oral Maxillofac Surg.

[15] Clementini M, Morlupi A, Agrestini C, Ottria L (2012). Success rate of dental implants inserted in autologous bone graft regenerated areas: a systematic review. Oral Implantol.

[16] Hollinger JO, Einhorn TA, Doll B, Sfeir C. Bone tissue engineering. Boca Raton: CRC Press; 2004. p. 352.

[17] Lynch SE. Tissue engineering: applications in oral and maxillofacial surgery and periodontics. Berlin: Quintessence Publishing Company; 2008.

[18] Demontiero O, Vidal C, Duque G (2012). Aging and bone loss: new insights for the clinician. Ther Adv Musculoskelet.

[19] Albrektsson T, Chrcanovic B, Östman P-O, Sennerby L (2017). Initial and long-term crestal bone responses to modern dental implants. Periodontology.

[20] Chrcanovic BR, Custódio ALN (2009). Inferior alveolar nerve lateral transposition. Oral Maxillofac Surg.

[21] Marx RE, Shellenberger T, Wimsatt J, Correa P (2002). Severely resorbed mandible: predictable reconstruction with soft tissue matrix expansion (tent pole) grafts. J Oral Maxillofac Surg.

[22] Poirot G, Delattre J, Palot C, Flament J (1986). The inferior alveolar artery in its bony course. Surg Radiol Anat.

[23] Castelli W (1963). Vascular architecture of the human adult mandible. J Dent Res.

[24] Mcgregor AD, MacDonald DG (1989). Age changes in the human inferior alveolar artery— a histological study. Brit J Oral Maxillofac Surg.

[25] Semba I, Funakoshi K, Kitano M (2001). Histomorphometric analysis of age changes in the human inferior alveolar artery. Arch Oral Biol.

[26] Karlis V, Bae RD, Glickman RS (2003). Mandibular fracture as a complication of inferior alveolar nerve transposition and placement of endosseous implants: a case report. Implant Dentistry.

[27] Losa PM, Cebrian JL, Guiñales J, Burgueño M, Chamorro M (2015). Mandibular fracture after inferior alveolar nerve lateralization: a rare and misunderstood complication. J Craniofac Surg.

[28] Haeberle CB, Abreu A, Metzler K, Robles-Moreno M. Complications associated with rehabilitation of a unilateral partially edentulous mandible with inferior alveolar nerve transposition and implant placement: a clinical report. J Prosthodont;n/a(n/a). [cited 2021 Feb 28]. Available from: 10.1111/jopr.13324 10.1111/jopr.13324 33434366

[29] Rahpeyma A, Khajehahmadi S (2019). Mandibular body fracture during inferior alveolar nerve transposition: review of literature. Ann Maxillofac Surg.

[30] Skalak R (1988). Stress transfer at the implant interface. J Oral Implantol.

[31] Loescher AR, Smith KG, Robinson PP (2003). Nerve damage and third molar removal. Dent Update.

